# ﻿Two new species, one new record, and one new synonym of *Aeschynanthus* (Gesneriaceae) from China

**DOI:** 10.3897/phytokeys.265.167339

**Published:** 2025-11-04

**Authors:** Lie-Wen Lin, Li-Yan Wang, Yong-Jie Guo, Lei Cai

**Affiliations:** 1 Yunnan Key Laboratory for Integrative Conservation of Plant Species with Extremely Small Populations, State Key Laboratory of Plant Diversity and Specialty Crops, Kunming Institute of Botany, Chinese Academy of Sciences, CN-650201, Kunming, Yunnan, China; 2 Kunming College of Life Science, University of Chinese Academy of Sciences, Kunming 650201, Yunnan, China; 3 Yingjiang Branch of Yunnan Tongbiguan Provincial Nature Reserve Administration, Yingjiang 676299, Yunnan, China; 4 Germplasm Bank of Wild Species, Kunming Institute of Botany, Chinese Academy of Sciences, Kunming, 650201, Yunnan, China

**Keywords:** *
Aeschynanthus
*, Flora of China, new synonym, new taxon

## Abstract

Southwest China is a biodiversity hotspot, with numerous new Gesneriaceae species recently discovered there. Recent reports of new *Aeschynanthus* taxa and records from western Yunnan and adjacent areas underscore the region’s continued potential for revealing undescribed diversity. Based on field surveys conducted in Yunnan and Xizang, Southwest China, two new species of *Aeschynanthus* are described: *A. succineus* Lei Cai & L.W. Lin and *A. tongbiguanensis* Lei Cai & L.W. Lin. We also report a new national record for China, *A. jouyi* D.J. Middleton, previously considered endemic to Vietnam. Furthermore, we propose that *A. maoi* Debta & A. Shenoy should be reduced to synonymy under *A. wardii*, based on a critical assessment of the literature, field observations of living plants, and examination of herbarium specimens. Descriptions and illustrations of the diagnostic characters are provided for these species, together with information on their types.

## ﻿Introduction

Southwestern China, encompassing the provinces of Yunnan and Xizang (Tibet), lies within the Indo-Burma Biodiversity Hotspot—one of the world’s most biologically significant regions (Brooks et al. 2006; Basnet et al. 2019). As a botanical center, it harbors flora spanning tropical, subtropical, temperate, and alpine zones, making it a critical reservoir of vascular plant diversity (Zhang et al. 2002; Yang et al. 2004). The region also functions as a major biogeographic transition zone, where the Sino-Himalayan, Southeast Asian, and East Asian floras converge. Levels of endemism are exceptionally high, with many species confined to narrow habitats such as limestone karsts, deep river valleys, and montane forests. This ecological and evolutionary complexity has made southwestern China a focal point for botanical research, with numerous new taxa of Gesneriaceae described in recent years. Its rugged topography and diverse hydrothermal conditions generate a wealth of microhabitats, providing ideal environments for the diversification and persistence of Gesneriaceae (e.g., Cai et al. 2019, 2020; Yang et al. 2019; Wang et al. 2022; Qin et al. 2023; Ding et al. 2024).

A notable example is the genus *Aeschynanthus* Jack, a group of evergreen, epiphytic, or lithophytic plants in the family Gesneriaceae. Species of *Aeschynanthus* typically grow on trees or rocks in moist, shaded forests, thriving under conditions of high humidity. Many are characterized by trailing or pendulous stems with often leathery leaves, and most produce tubular, brightly colored flowers—usually red or orange—adapted for bird pollination. For example, *A. acuminatus* is primarily pollinated by generalist birds (Chen et al. 2019). Beyond their ecological roles, several species are widely cultivated as ornamental houseplants, and some are also used in traditional folk medicine (Li et al. 2006). The genus comprises roughly 200 species distributed mainly across tropical and subtropical Asia, ranging from the Himalayas and Indochina Peninsula to New Guinea and the Solomon Islands (Wang et al. 1990, 1998; Middleton 2009, 2016). In China, 36 species have been documented (Wen et al. 2023), with more than 30 occurring in the border regions of southwestern China, underscoring the area’s importance as a center of diversity for the genus (Wang et al. 1990, 1998; Li and Wang 2005). In recent years, several new taxa and records of *Aeschynanthus* have been reported from western Yunnan, southeastern Xizang, and adjacent areas (e.g., Li et al. 2020; Qin et al. 2023; Ding et al. 2024; Debta et al. 2024; Dey et al. 2025), suggesting that this region remains a promising frontier for the discovery of undescribed species.

During recent field investigations in the Gaoligong Mountains and adjacent areas along the China–Myanmar border in Yunnan, China, we identified two distinctive species of *Aeschynanthus*. Comparison with national floras and relevant literature confirmed that these represent previously undescribed taxa, which are detailed below. In addition, based on our collections and field observations in western Yunnan, we propose that the recently published *A. maoi* should be reduced to synonymy under *A. wardii*. We also collected the showy species *A. jouyi* in the karst region of southeastern Yunnan, a taxon previously regarded as endemic to Vietnam. Accordingly, we describe two new species from China, report one new national record, and propose a new synonym under *A. wardii*.

## ﻿Materials and methods

Specimens of *Aeschynanthus* were collected during extensive field surveys conducted between 2015 and 2025 in various locations in Yunnan, including Gaoligong Mountain National Nature Reserve, Laoshan Provincial Nature Reserve, and Tongbiguan Provincial Nature Reserve. The following descriptions are based on both living collections and type specimens. We also examined material of *Aeschynanthus*, including digital images of specimens from 15 herbaria (PE, HITBC, KUN, IBSC, GXMG, IBK, GXMI, SZG, WUK, NAS, E, NY, P, K, BM) (Thiers 2025). Type specimens are deposited at KUN (Herbarium of the Kunming Institute of Botany, Chinese Academy of Sciences).

### ﻿Taxonomic treatment

#### ﻿ Aeschynanthus succineus

Taxon classificationPlantaeLamialesGesneriaceae

Lei Cai & L.W.Lin sp. nov.

3ED2ECCE-E443-55E4-9ACE-524D44E63CDC

urn:lsid:ipni.org:names:77371482-1

Fig. 1

##### Diagnosis.

*Aeschynanthus succineus* resembles *A. sinolongicalyx* and *A. longicaulis* in having pale green vein patterns on the leaves but can be distinguished by having longer tubulate flowers (35–45 mm vs 20.5–31 mm), fused calyx, and pseudoterminal cymes (vs solitary flower in leaf axils in *A. sinolongicalyx* and *A. longicaulis*). Most importantly, this new species can be distinguished from others by the dense pale-purple pubescence on the upper part of its style (Table 1).

**Table 1.** Morphological comparison of *Aeschynanthus succineus* sp. nov., *A. sinolongicalyx*, and *A. longicaulis* (Wang et al. 1990; Middleton 2009).

	A. succineus	A. sinolongicalyx	A. longicaulis
Inflorescence	cymes pseudoterminal, 2–11 flowers	cymes axillary, 1–3 flowers	cymes pseudoterminal, 1–3 flowers
Corolla	35–45 mm, yellow and orange	24–28 mm, red, yellow at base	20.5–31 mm, yellow-green
Calyx	16–22 mm, lobes fused below middle	25–35 mm, 5-lobed from base	8–18 mm, 5-lobed from base
Pistil	densely pubescent on the upper part	sparsely pubescent	sparsely pubescent

##### Type.

China • Yunnan Province, introduced from southwestern Yunnan (cultivated in Kunming Botanical Garden on 20 June, 2016, introduction code: KBG2017062005), in flower, 22 September 2020, *Cai Lei* CL2020092201 (Holotype: KUN!; Isotype: KUN! IBK!).

##### Description.

Epiphytic sub-shrubs, pendulous and branched. Stem glabrous, green, cylindrical. Leaves opposite; petiole 3–8 mm long, glabrous; leaf blade leathery, ovate or nearly lanceolate, 45–55 × 25–35 mm, weakly dentate at margins, glabrous on both sides, adaxial surface dark green with pale green vein patterns, abaxial surface pale green with pale red patterns. Cymes pseudoterminal, 2–11 flowers; pedicel glabrous, 6–15 mm long; bracts glabrous, 6–12 mm long, lanceolate. Calyx glabrous, greenish yellow to greenish red, 5-lobed, 16–22 × 4–6 mm, narrowly lanceolate, apex acute, margin entire, lobes fused into calyx tube (5–6 mm) at the base. Corolla 35–45 mm long, tubular, strongly oblique mouth, inflated at middle, externally yellow to orange, with 5 dark lines from the middle of corolla tube to the top of the lobes, glabrous or sparsely short-pubescent outside, with long purplish-red villous hairs robust hairs at the base of the corolla tube inside; lobes ca. 7 mm long, orange with purplish red spots at margin, apex rounded. Stamens 4, exserted; staminode ca. 1 mm long; anthers oblong, thecae parallel, pollen bright yellow. Filaments glabrous, cream white at the base, gradually changing to purplish red from the center to the top; anterior filaments 30–35 mm long, posterior filaments 22–28 mm long. Pistil 10–20 mm long, yellowish green, dense pale-purple pubescence on the upper part; ovary ca. 8 mm long, glabrous. Fruit and seed not observed.

**Figure 1.**
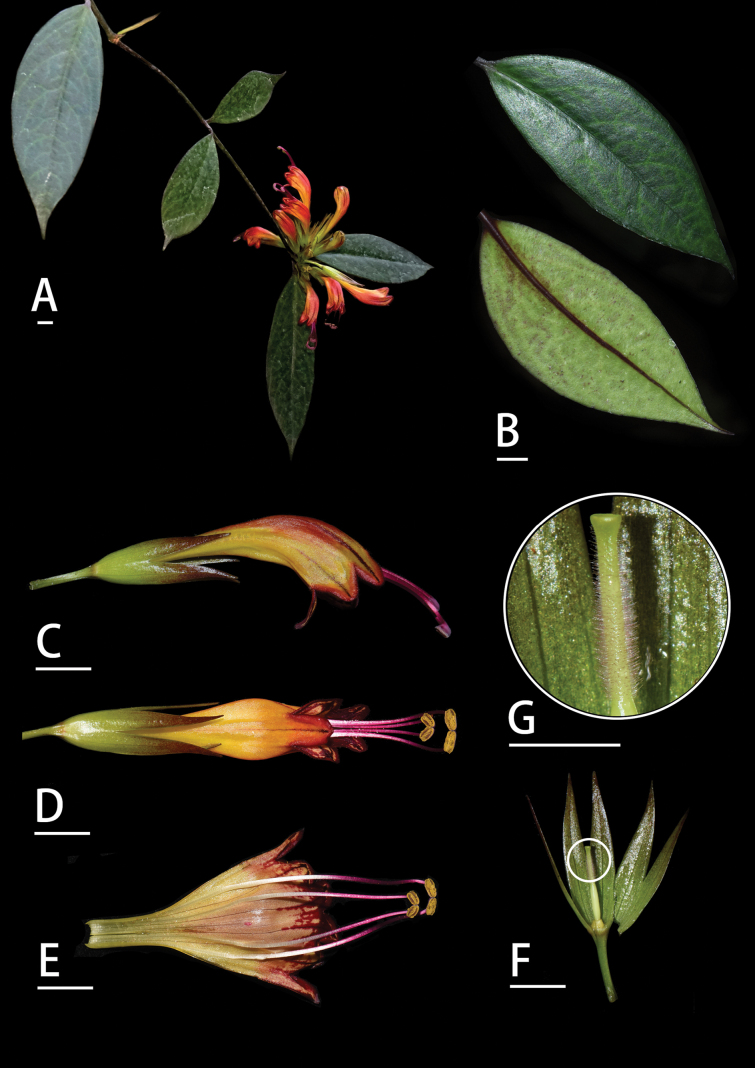
*Aeschynanthus succineus* Lei Cai & L.W. Lin, sp. nov. A. Flowering plant; B. Leaf surfaces; C. Flower in side view; D. Flower, abaxial side; E. Opened corolla; F. Calyx, ovary, and pistil; G. Close-up of pistil. Scale bars: 1 cm (photographed by Cai Lei).

##### Phenology.

Flowers from September to October.

##### Distribution and habitat.

According to the information provided by the cultivation staff, *Aeschynanthus succineus* is currently known only from material introduced from southwestern Yunnan and without detailed collected information. At present, this species is growing well on tree trunks in greenhouses of Kunming Botanical Garden.

##### Etymology.

This species bears warm-toned flowers ranging from yellow to orange, resembling amber in color. The specific epithet ‘*succineus*’ (Latin *succinum*, ‘amber’) reflects this distinctive hue.

##### Vernacular name.

琥珀芒毛苣苔 Hu Po Mang Mao Ju Tai.

##### Notes.

This species was described based on cultivated material introduced to a botanical garden, and no wild populations have yet been discovered. Its precise distribution range and population size remain unknown; therefore, its conservation status is assessed as Data Deficient (DD).

#### ﻿ Aeschynanthus tongbiguanensis

Taxon classificationPlantaeLamialesGesneriaceae

Lei Cai & L.W.Lin sp. nov.

F65B93B2-1CF6-53D9-A17C-E7838F342C41

urn:lsid:ipni.org:names:77371483-1

Fig. 2

##### Diagnosis.

This new species can be distinguished from *Aeschynanthus tubulosus* by its much smaller flowers (13–18 mm vs 30–35 mm) and leaves, along with lanuginose new shoots (vs pubescent young stems in *A. tubulosus*). Additionally, the glandular ovary and pistil, together with the included stamens and stigma of *A. tongbiguanensis*, are absent in other shrub-like *Aeschynanthus* species in the region (such as *A. buxifolius*, *A. andersonii*, and *A. humilis*). Moreover, the flower phenology of *A. tongbiguanensis* does not overlap with these other species (Table 2).

**Figure 2.**
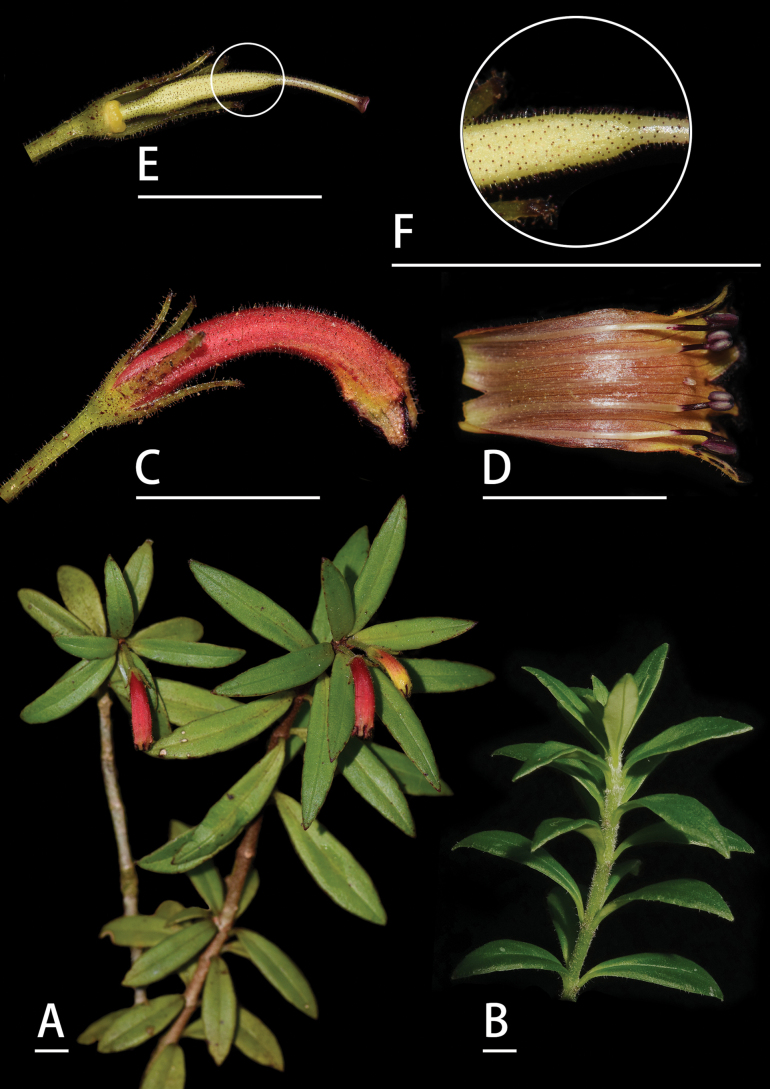
*Aeschynanthus tongbiguanensis* Lei Cai & L.W. Lin, sp. nov. A. Flowering plant; B. Young shoot; C. Flower in side view; D. Opened corolla; E. Calyx, ovary, and pistil; F. Close-up of pistil. Scale bars: 1 cm (photographed by Cai Lei and Lin Lie Wen).

**Table 2.** Morphological comparison of *Aeschynanthus tongbiguanensis* sp. nov., *A. tubulosus*, and *A. humilis* (Wang et al. 1990; Middleton 2009).

	A. tongbiguanensis	A. tubulosus	A. humilis
Leaf blade	oblanceolate to spatulate	elliptic to ovate	oblanceolate to spatulate
Inflorescence	cymes pseudoterminal, 1–3-flowered	cymes axillary, 1-flowered	cymes pseudoterminal, 1–4-flowered
Corolla	13–18 mm, outside glandular	30–35 mm, outside glabrous	15–28 mm, outside sparsely puberulent
Calyx	6–8 mm, outside glandular	5–9 mm, outside glabrous	1–4.2 mm, outside puberulent
Pistil	included, glandular	exserted, glabrous	exserted, puberulent
Stamens	included, nearly equal	exserted, strongly dimorphic	exserted, strongly dimorphic
Flower phenology	Dec–Feb	March	Sept–Oct

##### Type.

China • Yunnan Province, Dehong Dai and Jingpo Autonomous Prefecture, Yingjiang County, Tongbiguan town, Banggetong, 24°36'16.55"N, 97°39'02.74"E, ca. 1298 m, 20 Dec 2024 (fl.), *Cai Lei & Wang Li-Yan CL622* (holotype: KUN!; isotypes: KUN! IBK!).

##### Description.

Epiphytic small shrubs, erect and branched. Young stem green, lanuginose; the second-year branches brown, manicate. Leaves opposite or 3-verticillate; petiole ca. 3 mm long; leaf blade leathery, oblanceolate to spatulate, 7–25 mm × 6–12 mm, weakly dentate at margins, sparsely ciliate, adaxial surface green, abaxial surface pale green, glabrous on both surfaces, lateral veins obscure. Cymes pseudoterminal, 1–3 flowers; pedicel, calyx, abaxial petal surface, ovary, and style are all covered with dark glandular trichomes; pedicel 8–15 mm, greenish yellow with purplish spots; calyx greenish yellow, 6–8 mm, 5-lobed from below middle, narrowly lanceolate, apex acute. Corolla 13–18 mm long, tubular, strongly oblique mouth, inflated at middle, externally red or reddish orange, abaxial surface orange-yellow, entirely glabrous except for sparsely papillae near base, with purplish red spots at margin; lobes ca. 3 mm long, erect, neither reflexed nor spreading, apex rounded. Stamens and stigma included. Stamens 4, staminode ca. 1 mm long; anthers oblong, pollen pale yellow. Filaments nearly equal, glabrous, creamy yellow at the base, gradually changing to purplish red at the top. Pistil ca. 5 mm long, yellowish green; ovary 6–8 mm long. Fruit and seed not observed.

##### Phenology.

Flowers from December to February (the following year).

##### Distribution and habitat.

At present, *Aeschynanthus tongbiguanensis* is known only from its type locality in Tongbiguan, Yingjiang County, China, where it grows on tree trunks in evergreen forests at an elevation of ca. 1300 m.

##### Etymology.

The specific epithet ‘tongbiguanensis’ refers to the type locality where the new species was found, Tongbiguan Township, Yingjiang County, Yunnan Province, China.

##### Vernacular name.

铜壁关芒毛苣苔 Tong Bi Guan Mang Mao Ju Tai.

##### Notes.

**Figure 3.**
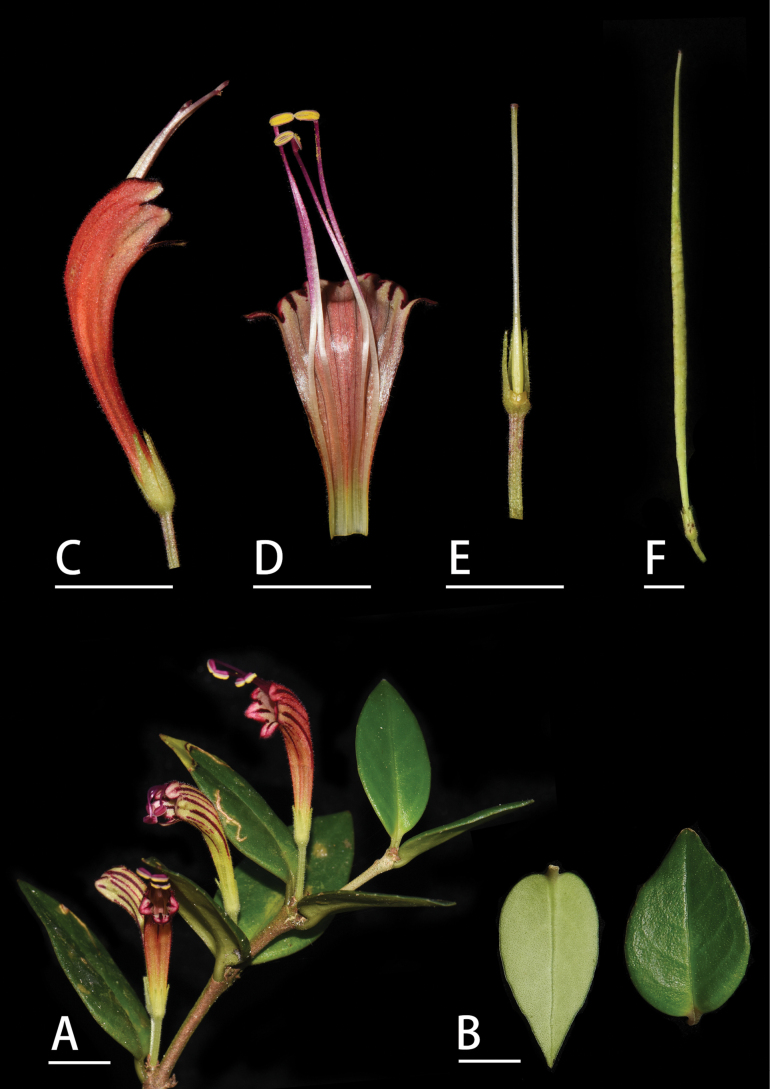
*Aeschynanthus tubulosus* J. Anthony. A. Flowering plant; B. Leaf surfaces; C. Flower in side view; D. Opened corolla; E. Calyx, ovary, and pistil; F. Fruit. Scale bars: 1 cm (photographed by Cai Lei and Lin Lie Wen).

Two epiphytic, shrub-like *Aeschynanthus* species were collected from adjacent sites in Tongbiguan Town, Yunnan Province, China. We confirmed one as the known species *Aeschynanthus tubulosus* (Fig. 3), while the other represents a previously undescribed species, which we formally describe here as *Aeschynanthus tongbiguanensis*. Currently, only about a dozen individuals have been observed on a few trees, with the overall population status remaining unclear. Therefore, its conservation status is assessed as Data Deficient (DD).

### ﻿New records to China

#### ﻿ Aeschynanthus jouyi

Taxon classificationPlantaeLamialesGesneriaceae

D.J.Middleton

4F415523-4EDF-5D3C-A92F-2CD038DBF0CE

Fig. 4

Aeschynanthus jouyi D.J.Middleton, Edinb. J. Bot. 66 (3): 417. 2009.

##### Type.

Vietnam • Lao Cai, Van Ban District, Liem Phu, Hoang Lien Mountain Range, Ta Xa Mountain close to the Nam Qua River, *Northern Vietnam First Darwin Expedition, #105* (holotype: E [barcode E00294857]; isotype: P [barcode P00697843]).

##### Distribution and habitat.

In China, *Aeschynanthus jouyi* is found in Malipo County, Yunnan, where it grows on trees in broad-leaved forests at 800–1000 m near the Sino–Vietnam border. It is also distributed in montane forests of northern Vietnam at 450–1500 m.

##### Vernacular name.

中越芒毛苣苔 Zhong Yue Mang Mao Ju Tai.

##### Specimens examined.

China • Yunnan Province, Malipo County, Xiajinchang Township, Huangtian Village (cultivated in Kunming Botanical Garden), 28 Jun 2018 (fl), *Cai Lei CL2018062801* (KUN!); • Yunnan Province, Malipo County, Tianbao Township, Kangjiatang, 23°1'8.9"N, 104°49'25.8"E, ca. 1200 m, 13 August 2019 (fl), *Ya Ji-Dong & Zhang Wei 19CS18574* (KUN!).

##### Description.

Epiphyte with robust arching and pendulous stems; stems green, glabrous. Leaves opposite; petiole 1.5–3 mm long, green, glabrous; blade coriaceous, elliptic, dark green above, paler beneath, occasionally reddish-tinged, not marbled, 3.5–6.5 × 8–13 cm, apex acuminate, base acute to cuneate, glabrous above and beneath, punctate beneath, margin entire to weakly crenate towards apex, c.4 pairs of secondary veins, weakly visible. Inflorescence terminal, 2–7-flowered; peduncle 2–13 mm long; pedicels 12–21 mm long, green, glabrous. Calyx with a long tube and free lobes, tube not clasping corolla and erect at top, green or maroon, glabrous, 28–35 mm long; tube 18–25 mm long; lobes narrowly triangular, erect, 8–11 × 2.8–4 mm, apex acute. Corolla 7.5–8.5 cm long, tube broad at base, externally bright red, internally pale red, lobes bright red, internally red with darker markings on lower 3 lobes; upper lobes oblong, not spreading or reflexed, almost appearing as a single lobe, apex rounded; lateral lobes oblong, spreading to reflexed; lower lobe oblong, spreading to reflexed, apex rounded to truncate; outside with small glandular hairs, inside with scattered glandular hairs throughout except at base. Stamens slightly exserted, fused in 2 pairs; filaments pale orange at base and red higher, sparsely glandular pubescent, anthers orange; anterior filaments inserted, 3.0–4.5 cm long; staminode c.2.5 mm long. Pollen ochre. Disk 5-crenate. Pistil c.7.5 cm long, glabrous; ovary pale green, c.26 mm long, glabrous; style c.11.5 mm long, glabrous. Fruit unknown.

**Figure 4.**
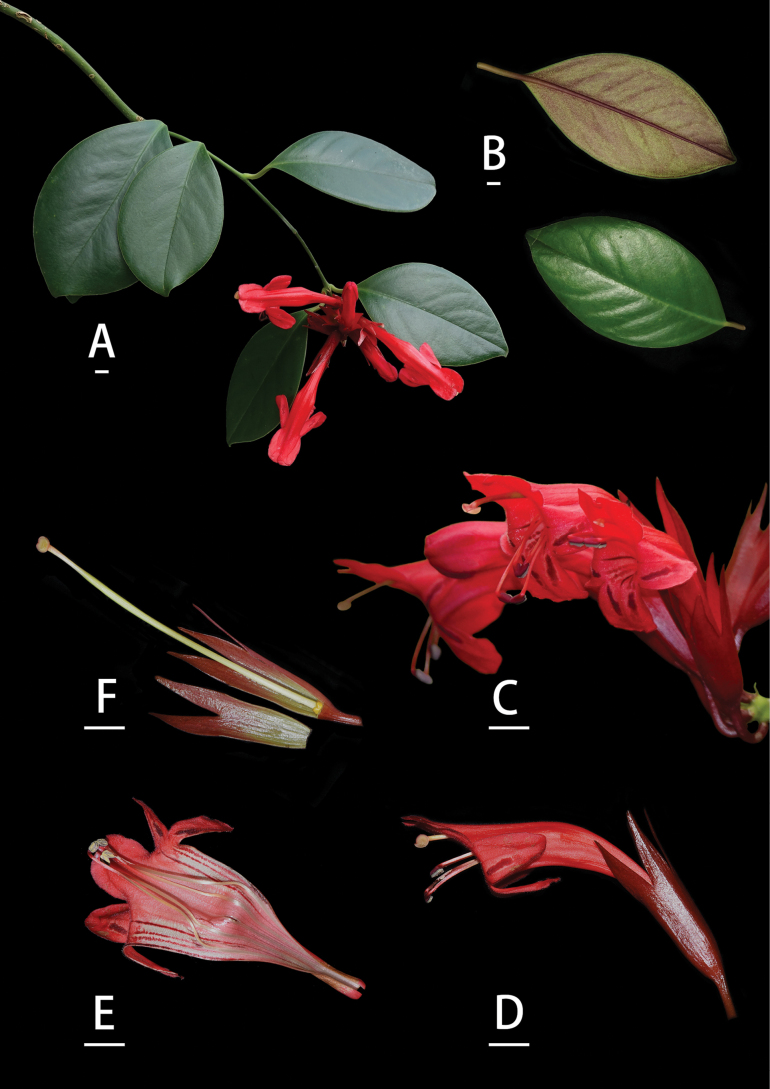
*Aeschynanthus jouyi* D.J. Middleton. A. Flowering plant; B. Leaf surfaces; C. Flower in front view; D. Flower in side view; E. Opened corolla; F. Calyx, ovary, and pistil. Scale bars: 1 cm (photographed by Cai Lei and Lin Lie Wen).

##### Phenology.

Flowering from June to August.

##### Notes.

The type collection is taken from a plant in the greenhouse at the Royal Botanic Garden Edinburgh, which was originally collected from Lao Cai Province, northern Vietnam (Middleton, 2009). We recently collected this species in Malipo County along the China-Vietnam border. While the type specimens and original description characterize the leaves as pale green abaxially with green calyx, the specimens we collected in China exhibit maroon calyx and occasionally reddish-tinged leaf undersurfaces, with no other significant morphological differences.

### ﻿New synonyms

#### ﻿ Aeschynanthus wardii

Taxon classificationPlantaeLamialesGesneriaceae

Merr., Brittonia 4: 173. 1941.

4EBE3992-CE85-5619-AF20-D49BB1A212A2

Fig. 5

Aeschynanthus maoi Debta & A.Shenoy, Brittonia 76:162 (2024), syn. nov. Type. India: Arunachal Pradesh, Lower Dibang Valley district, near Hunli, 28°20'09.4"N, 95°56'48.4"E, ca. 1,190 m, 18 Oct 2022 (fl.), *M.R. Debta & A. Shenoy 44200* (holotype: ARUN, barcode ARUN000030895; isotypes: ARUN, barcode ARUN000030893; CAL; CALI).

##### Type.

Upper Burma or southeastern Tibet • location not indicated, 4000–5000 ft, 22 Nov. 1931, *F. Kingdon-Ward, 10170* (isotype: BM [barcode BM000883858]; A [barcode A00057319]).

##### Distribution and habitat.

W & NW Yunnan and SE Xizang (China); also N Myanmar and NE India. It grows on the surfaces of trees or stones under broad-leaved forests at 800–2500 m.

##### Taxonomic discussion.

**Figure 5.**
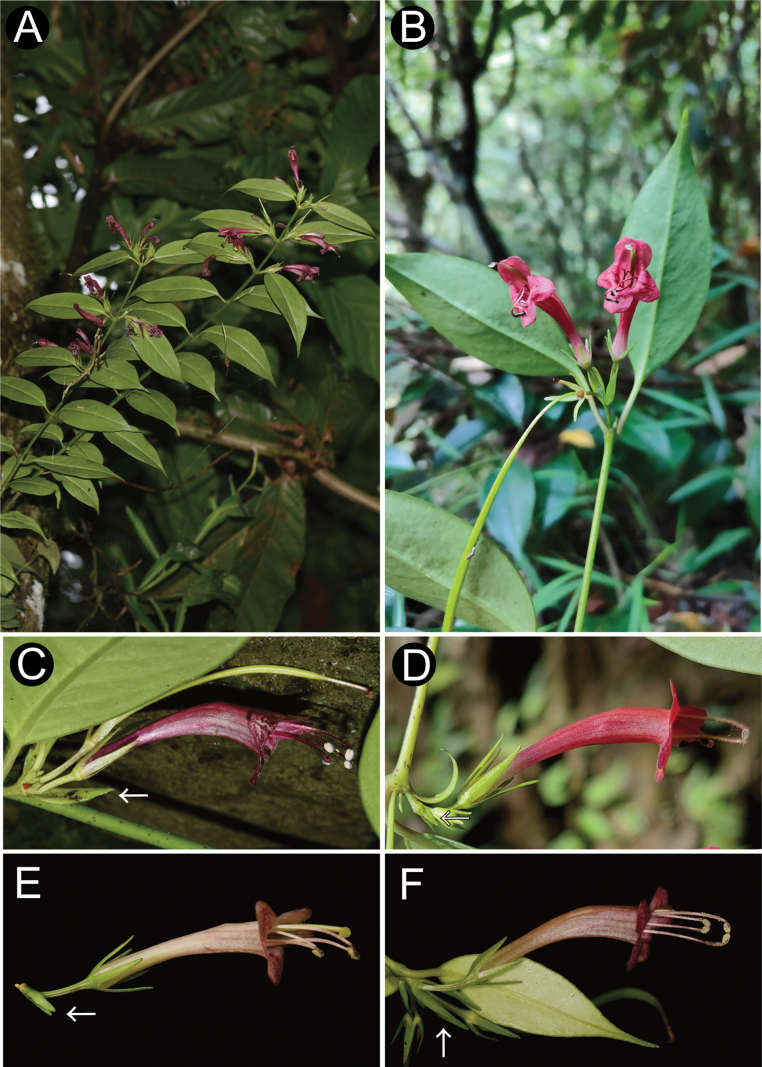
*Aeschynanthus wardii* Merr. A–B. Plant in flowering; C–F. Flowers in side view. The white arrow represents 0.5 cm. (A, C: photographed by Guo Shi-Wei in Lushui; B, E, F: photographed by Guo Yong-Jie in Yingjiang; D: photographed by Liu Cheng in Fugong).

*Aeschynanthus wardii* was described from southeast Xizang or Upper Burma in the original description in 1931 (with doubt over the exact location expressed in the protologue) and later found in Yunnan Province, China (Li 1983; Merrill 1941). In 2022, Debta et al. collected an *Aeschynanthus* species in the southern slope region of the Himalayas and described it as *A. maoi* (Debta et al. 2024). The authors proposed that *A. maoi* can be distinguished from *A. wardii* by differences in bract shape/size, calyx lobe morphology, and flower coloration. However, these two species show certain similarities in many morphological features, particularly in vegetative parts and corolla shape. Based on extensive field observations, we noticed that bract and calyx traits, as well as flower color, exhibit substantial intraspecific variation (Fig. 5), rendering these features unreliable for species delimitation. Moreover, the Gaoligong Mountains in Yunnan, southeastern Xizang, northern Myanmar, and parts of northeastern India share similar climates and vegetation. In our previous field investigations, we frequently encountered numerous species distributed across national borders. After carefully reviewing the relevant high-definition ink lines and color image information in the article and considering the normal population variation of this species, we suggest that *A. maoi* should be reduced to a synonym of *A. wardii*.

##### Additional examined specimens.

China • Yunnan Province. Fugong County, on the way from Shiyueliang Township to Yaping, 27.1462519 N, 98.8177871 E, alt. 2040 m, 21 October, 2024, *Liu Cheng et al. 24CS27228* (KUN!); • Yingjiang County, Zhina Township, on the surface of the tree trunk, 25°12'16.38"N, 98°2'47.19"E, alt. 1535 m, 26 September, 2024, *Guo Yong-Jie et al. 24CS26803* (KUN!); • Yingjiang County, Zhina Township, Zhidong Village, Dazhupeng, on stone surfaces besides a river, 25°15'8.08"N, 98°6'44.26"E, alt. 1327 m, 26 October, 2023, *Guo Yong-Jie et al. 23CS25289* (KUN!); • Lushui City, Liuku Township, Pailuba Village, Achidahe, on stone surfaces, 25°50'56.04"N, 98°50'56.04"E, alt. 1697 m, 11 October, 2019, *Guo Shi-Wei et al. KIBDZL212B02* (KUN!); • Gongshan County, Dulongjiang Township, alt. 1500–1600 m, 8 August, 1982, Qing Zang Dui, 9074 (PE); • Jingdong County, Wuliangshan, alt. 2300 m, 7 November, 1956, *Qiu Bing-Yun et al.*, *53453* (PE); • Monting, Kiukiang Valley, alt. 1350 m, 24 September, 1938, *T.T.Yu et al. 20410* (PE); • Shang-pa, alt. 2800 m, 10 October, 1934, *H.T.Tsai et al. 58702* (IBSC); • Shang-pa, 10 October, 1933, *H.T.Tsai et al. 58702* (PE); • Shang-pa, alt. 2000 m,18 September, 1933, *H.T.Tsai et al. 54336* (PE); • Hills to the Northwest of Tengyueh, alt. 1666 m, January, 1913, *George Forrest et al. 9499* (PE).

##### Description.

Stems 1–2 m, glabrous. Leaves opposite; petiole 0.7–1.5 cm; leaf blade narrowly elliptic to ovate or obovate, 5.5–10 × 1.6–3.9 cm, leathery to papery, glabrous, adaxially drying smooth, abaxially few punctate, base cuneate to rounded, margin entire to shallowly crenulate, apex caudate to acuminate; lateral veins indistinct. Cymes axillary, 1–4 (–10)-flowered; peduncle absent to 0.5 (–1) cm; bracts persistent or deciduous, green, lanceolate to narrowly ovate, 5–18 × 2–7 mm. Pedicel 4–10 mm, glabrous. Calyx green, 5–sect from base; segments lanceolate–linear, 7–9 × 1.1–1.5 mm, outside glabrous. Corolla color highly variable: red, orange-red, purplish-red, wine-red, or nearly flesh-colored, ca. 3.5 cm, outside glabrous, inside sparsely glandular puberulent below abaxial lip, without tufts of hairs, mouth oblique; limb indistinctly 2-lipped; adaxial lip erect, ca. 4.5 mm; abaxial lip reflexed, ca. 4.5 mm. Stamens exserted; filaments 1.4–2 cm; anthers coherent in pairs at apex, 1.5–2 mm; staminode ca. 0.6 mm. Pistil ca. 3.5 cm; ovary sparsely glandular puberulent. Style ca. 7 mm, sparsely glandular puberulent. Capsule 7–10 cm. Seeds with 1 hairlike appendage at each end, appendages 1.5–2.5 mm.

##### Phenology.

Flowering from June to December.

##### Vernacular name.

狭花芒毛苣苔 Xia Hua Mang Mao Ju Tai.

## Supplementary Material

XML Treatment for Aeschynanthus succineus

XML Treatment for Aeschynanthus tongbiguanensis

XML Treatment for Aeschynanthus jouyi

XML Treatment for Aeschynanthus wardii

## ﻿Additional information

### Use of AI

No use of AI was reported.

### Author ORCIDs

Lie-Wen Lin https://orcid.org/0000-0003-0925-2581

Yong-Jie Guo https://orcid.org/0000-0001-5541-572X

Lei Cai https://orcid.org/0000-0002-9251-2745

### Data availability

All of the data that support the findings of this study are available in the main text.
